# Hmo1 Promotes Efficient Transcription Elongation by RNA Polymerase I in *Saccharomyces cerevisiae*

**DOI:** 10.3390/genes15020247

**Published:** 2024-02-15

**Authors:** Abigail K. Huffines, David A. Schneider

**Affiliations:** Department of Biochemistry and Molecular Genetics, University of Alabama at Birmingham, Birmingham, AL 35294, USA; mcconaha@uab.edu

**Keywords:** RNA polymerase I, high mobility group protein 1, rRNA

## Abstract

RNA polymerase I (Pol I) is responsible for synthesizing the three largest eukaryotic ribosomal RNAs (rRNAs), which form the backbone of the ribosome. Transcription by Pol I is required for cell growth and, therefore, is subject to complex and intricate regulatory mechanisms. To accomplish this robust regulation, the cell engages a series of trans-acting transcription factors. One such factor, high mobility group protein 1 (Hmo1), has long been established as a trans-acting factor for Pol I in *Saccharomyces cerevisiae*; however, the mechanism by which Hmo1 promotes rRNA synthesis has not been defined. Here, we investigated the effect of the deletion of *HMO1* on transcription elongation by Pol I in vivo. We determined that Hmo1 is an important activator of transcription elongation, and without this protein, Pol I accumulates across rDNA in a sequence-specific manner. Our results demonstrate that Hmo1 promotes efficient transcription elongation by rendering Pol I less sensitive to pausing in the G-rich regions of rDNA.

## 1. Introduction

In eukaryotes, there are at least three RNA polymerases (Pols), which are required for transcribing unique DNA targets into RNA. Pol I synthesizes the 18 S, 5.8 S, and 25 S ribosomal RNAs (rRNAs), Pol II synthesizes messenger RNA (mRNA), and Pol III synthesizes transfer RNAs (tRNAs) and the 5 S rRNA. Transcription factors are essential for efficient RNA synthesis and are critical for regulating the unique activities of the three Pols. These factors promote initiation, elongation, and/or termination and are often exclusive to one or sometimes two of the three Pols. In fact, out of the myriad of known transcription factors, only one, TATA-binding protein (TBP), is part of the transcription machinery of all three Pols [1]. This diverse collection of transcription factors demonstrates the complexity of the regulatory mechanisms that govern RNA synthesis.

One superfamily of transcription factors, high mobility group (HMG) proteins, is conserved and found in abundance throughout eukaryotes [2]. This superfamily is composed of three subfamilies, HMGA, HMG-Box (HMGB), and HMGN, which are distinguished by their different functional motifs [3,4,5,6]. The proteins in the HMG-Box subfamily, which is the largest of the three, contain at least one HMGB that facilitates their DNA-binding properties [7,8]. Upon binding, these proteins are thought to mediate DNA organization, possibly by inducing looping and bending [9]. One such protein in *S. cerevisiae* (yeast) is high mobility group protein 1 (Hmo1), which has been identified to be part of the transcription machinery for both Pols I and II. Although Hmo1 is exclusively found in yeast [10], it is considered to be the functional analog of upstream binding factor (UBF) in humans due to its partial sequence conservation and shared localization to the nucleolus (the location of transcription by Pol I) in their respective species [11]. UBF is an important component of the pre-initiation complex and promotes the maintenance of accessible active ribosomal DNA (rDNA) repeats [12,13,14,15]. Similar to UBF, previous literature has demonstrated that Hmo1 localizes to rDNA and the promoter region of genes that encode for ribosomal proteins, indicating that Hmo1 plays a role in ribosome biogenesis [16]. However, it is still unclear whether the mechanism by which Hmo1 activates transcription in yeast is the same as UBF in humans.

Since its initial discovery [17], the precise mechanism of transcriptional activation by Hmo1 has remained elusive. However, a recent study demonstrated that Hmo1 may regulate transcription by Pol II through coordination with Topoisomerase 1 (Top1) and Topoisomerase 2 (Top2; [18]). Topoisomerases, including Tops 1 and 2, are highly conserved proteins that alleviate and resolve DNA supercoiling, which naturally occurs during replication and transcription [19]. The results of that study showed that Hmo1 and Top2 occupy the same region of Pol II-transcribed genes (outside of the open reading frame) and that this region is associated with negative supercoiling. Likewise, they determined that in both *hmo1Δ* and *top2-ts* mutants, there was a decrease in negative supercoiling in these regions. Finally, their findings indicated that Tops 1 and 2 could partially compensate for each other since Top1 was localized to the normal binding range for Top 2 in *top2-ts* mutants. Collectively, these results suggest that the interplay between Hmo1 and Tops1 and 2 is important for resolving positive supercoiling and maintaining a negatively supercoiled state in the yeast system, thus promoting efficient transcription. Furthermore, it has been shown that the mammalian HMGB protein, HMGB1, interacts with the human topoisomerase topoIIα, suggesting that this mechanism could be conserved throughout eukaryotes [20].

If rDNA topology is influenced by interactions between topoisomerases and Hmo1, then we predict that there could be an effect on transcription elongation by Pol I in yeast cells lacking *HMO1* (*hmo1Δ*). To test this, we used native elongating transcript sequencing (NET-seq), which allows for the mapping of polymerase occupancy (global positioning) at single-nucleotide resolution. Based on what has been previously described for Pol II, we hypothesized that without Hmo1, the efficiency of transcription elongation by Pol I would be reduced. Using this technique, we determined that in *hmo1Δ* yeast, Pol I occupancy is significantly altered across the rDNA, especially at the 3′ end of the 35 S gene, in a sequence-specific manner. This study demonstrates that Hmo1 is an important transcription elongation factor, and without it, Pol I is significantly more prone to pausing during rRNA synthesis.

## 2. Materials and Methods

### 2.1. Native Elongating Transcript Sequencing (NET-seq)

Two strains of yeast, wild-type ((His)7-(HA)3-*RPA135::TRP1*) and *hmo1Δ* ((His)7-(HA)3-*RPA135::TRP1, hmo1Δ*::*URA4*), were used for this study. NET-seq samples were generated in triplicate for each strain. Experiments were performed exactly as previously described [21,22,23,24,25]. In brief, one liter per replicate (three replicates per strain; six liters in total for the entire study) was grown using YEPD at 30 °C with nutation until reaching an OD600 of 0.3. Cells were rapidly harvested via filtration and lysed under cryogenic conditions using the same technique as described in previous publications. After lysis, anti-HA beads were blocked overnight at 4 °C, and immunoprecipitation for Pol I was performed according to previously published methods. The total RNA was extracted with acidic phenol (pH 4.3) and chloroform. After the extraction, a unique molecular identifier (UMI; [21,22,23,24,25]) containing DNA oligo was ligated onto the 3′ end of the extracted RNAs. Next, reverse transcription was performed, and DNAs between 120 and 600 bp were excised and extracted from 10% polyacrylamide gel. The gel-extracted DNA was circularized, and the libraries were amplified (primer sequences are included in Table 1). Finally, libraries were prepared for sequencing with PCRCLEAN DX beads by following the manufacturer’s suggested protocol. Reads were sequenced using an Illumina NextSeq 500 sequencer with the same sequencing primer as described previously [21,22,23,24,25].

### 2.2. NET-seq Data Processing and Analysis

After sequencing, libraries were processed and analyzed exactly as previously described [21,22,23,24] with software and versions included in Table 2. First, the read quality and quantity for each sample were assessed with FastQC. Fqtrim was used to reduce PCR bias by deduplicating reads based on their UMI sequence, and cutadapt was used to remove the 5′ and 3′ adaptor sequences. Alignment to the yeast genome (*S. cerevisiae* assembly 64-1-1) was conducted with STAR. After alignment, output BAM files were sorted, indexed, and converted to BED files with BEDTools. The resulting BED files were used to generate genome coverage files containing the chromosome, position, and counts at each position. These files were imported into RStudio for visualization of the data via R.

### 2.3. NET-seq Data Visualization

**Figure 1:**
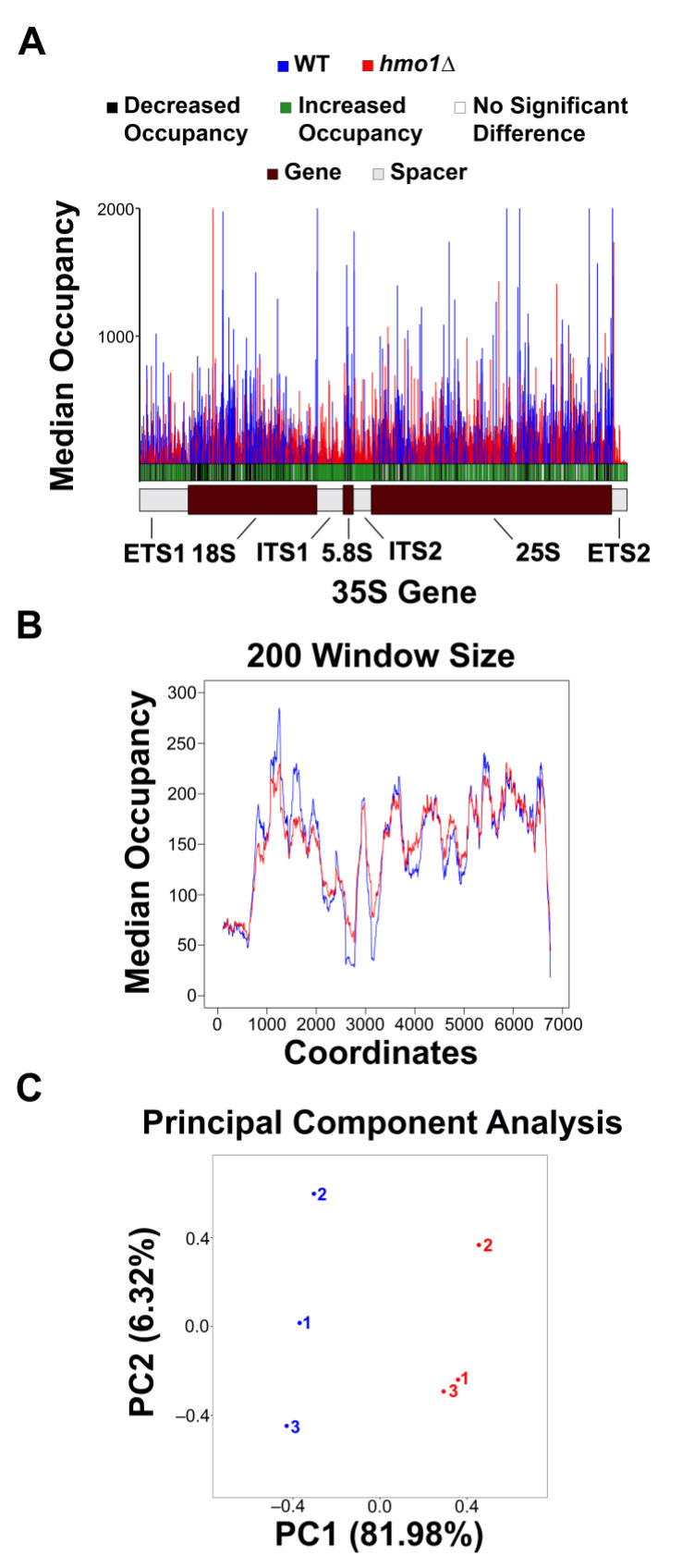
**Pol I occupancy patterns in WT vs. *hmo1Δ* strains.** (**A**) The median Pol I occupancy was plotted for WT (blue) and *hmo1Δ* (red) yeast. Student’s *t*-test was performed, where a significant difference was deemed to be indicated by a *p*-value < 0.05. For *hmo1Δ* with respect to WT, when significance (*p*-value < 0.05) was detected, an increase was recorded as a green line, and a decrease was recorded as a black line in the significance bar directly below the histogram. (**B**) The moving average across a 200-nucleotide window for the WT and *hmo1Δ* strains. (**C**) Principal component analysis was performed and plotted for all the replicates included in this study.

**Figure 2:**
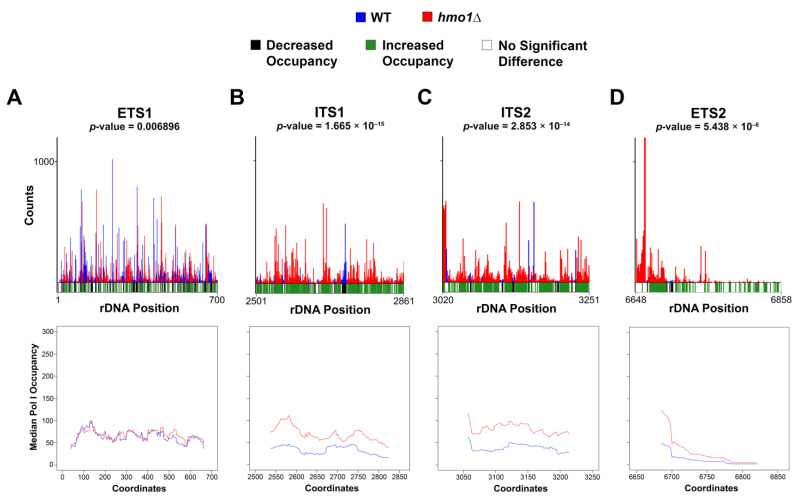
**Spacer region analysis indicates increased Pol I pausing in cells lacking *HMO1*.** Similar to Figure 1A, the median Pol I occupancy across the four spacer regions ((**A**) ETS1, (**B**) ITS1, (**C**) ITS2, and (**D**) ETS2) was plotted via histograms for the WT and *hmo1Δ* strains. As described in Figure 1, a significance bar is included below each histogram, which contains the results from the Student’s *t*-test (green or black represents a *p*-value < 0.05; a green line indicates a significant increase; and a black line indicates a significant decrease in the *hmo1Δ*vs. WT strain). Resulting *p*-values from the Kolmogorov–Smirnov test are included directly above each plot. A moving average plot with a window size of 75 was generated for each spacer region (bottom panels).

**Figure 3:**
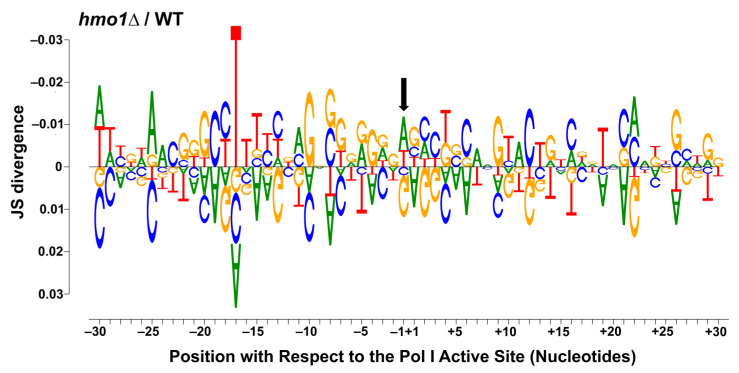
**DiffLogo indicates the repositioning of Pol I in *hmo1Δ* yeast.** A DiffLogo was generated to compare the top 2.5% highest occupied sequences in the spacer regions only for the *hmo1Δ* (top, JS divergence > 0) and WT (bottom, JS divergence < 0) strains. The DiffLogo is centered on the last incorporated nucleotide, which is identified by the black arrow.

Visualized data (represented in Figure 1, Figure 2 and Figure 3 and Supplementary Figure S1) were normalized by dividing the number of counts at each position by the total number of counts for that sample. Data were organized and compiled into a data frame containing the coordinate, 35 S region, normalized individual counts, normalized mean and median counts, and statistical analysis results (included in GEO submissions, accession information below). Initial histograms comparing the Pol I occupancy patterns between replicates within the same strain (Figure 1), as well as the moving average plots (Figure 1B and Figure 2), were generated via the built-in plotting function of R (with the installation of the RcppRoll package for the moving average plots). The median comparison histograms for Pol I occupancy (Figure 1A and Figure 2) were created with ggplot2. The principal component analysis (Figure 1C) was conducted and visualized with ggfortify. Finally, the sequence enrichment analysis in Figure 3 was generated using the DiffLogo package. All statistical analyses (Spearman correlation test and Kolmogorov–Smirnov test) were performed using R. Raw and processed files are included on the Gene Expression Omnibus database, with the WT files available at GSE216460 and the *hmo1Δ* files available at the GSE247981 accession. R scripts used for the visualization of data and the generation of all figures are available upon request.

## 3. Results

### 3.1. Pol I Occupancy Is Increased in hmo1Δ Yeast

To test whether Hmo1 promotes Pol I transcription elongation, we deployed NET-seq [26] to investigate Pol I occupancy patterns at single-nucleotide resolution [21,22,23,24,25,27]. Triplicate samples were prepared, libraries were generated for both WT and *hmo1Δ* yeast, and the reads mapping to the 35 S rDNA gene were isolated and plotted. The data were normalized by dividing the number of reads at each single-nucleotide position by the total read count for each individual replicate. These normalized counts were used to generate all figures included in this study. Histograms (visualized as individual replicates (left panels) and as an overlay of all three replicates (middle panels)) and Spearman correlation coefficient values (right panels; all comparisons > 0.9) indicated that Pol I occupancy patterns were highly reproducible across replicates within the same strain (Supplementary Figure S1).

After determining that NET-seq was reproducible between replicates for both of the strains analyzed in this study, we next used this tool to compare occupancy patterns between WT and *hmo1Δ* yeast. For each strain, the median Pol I occupancy across the three replicates was plotted and overlaid (Figure 1A). To probe for significant differences between strains, a Student’s *t*-test was performed at every single-nucleotide position. If a significant difference was detected (*p*-value < 0.05), this was indicated with a green line for an increase or a black line for a decrease in the *hmo1Δ* vs. WT strain. These results are included in the bar (the significance bar) below the median occupancy histogram (Figure 1A). The resultant data display a somewhat periodic pattern for both strains (consistent with the previously published crosslinking and analysis of cDNA (CRAC) data [28]). Interestingly, the abundance of black and green in the significance bar indicates that Pol I occupancy is significantly altered at the majority of positions in the 35 S gene in *hmo1Δ* yeast vs. WT. Furthermore, there are peaks of high occupancy in the WT profile that are lower in the *hmo1Δ* strain and vice versa. While we cannot deduce kinetic information from NET-seq results due to the lack of a time variable, the simplest interpretation of NET-seq data is that high peaks represent Pol I pausing, while low peaks represent more rapid transcription by Pol I. Therefore, Figure 1A indicates that Pol I pause sites are modified in *hmo1Δ* yeast vs. WT. These patterns were confirmed by plotting the moving average of the median occupancy between strains (Figure 1B) and by performing a principal component analysis (PCA) between the samples (Figure 1C). Altogether, from these results, we conclude that Hmo1 promotes efficient transcription elongation and the resolution of Pol I pausing either directly or indirectly.

In previous publications, we demonstrated that Pol I NET-seq libraries contain mature rRNA contamination [23,24]. Therefore, an in-depth analysis of the spacer regions (ETS1, ITS1, ITS2, and ETS2) is critical to focus exclusively on the nascent RNA signal. To investigate Pol I occupancy in the spacers, the median occupancy and the moving average of the median occupancy (using a window size of 75 positions) were analyzed and plotted for each strain in the four spacer regions (Figure 2A–D), the same way as described in Figure 1A,B. For each region, we deployed the Kolmogorov–Smirnov (K-S) test, which probes for significant differences in the distribution patterns between strains (resultant *p*-values are included above each panel; Figure 2A–D). Overall, we observed that in both strains, there are peaks and valleys of Pol I occupancy, which are significantly different in relation to each other in all four spacer regions, confirming the altered pause profile in the *hmo1Δ* vs. WT strain (as observed in Figure 1). Furthermore, these occupancy pattern changes were exacerbated in the ITS1, ITS2, and ETS2 regions (the middle and the 3′ ends of the 35 S gene) in *hmo1Δ* vs. WT yeast. Altogether with Figure 1, these results suggest that Hmo1 is an important transcription elongation factor for Pol I and may promote efficient topology maintenance throughout the rDNA, which is consistent with the proposed function of this factor for Pol II [18].

### 3.2. Pol I Is Sensitive to T/G-Rich Regions in hmo1Δ Yeast

Based on the median occupancy histograms and spacer analysis (Figure 1 and Figure 2), we determined that the Pol I occupancy patterns were altered in the *hmo1Δ* strain compared to WT. Therefore, we next investigated whether this altered pause propensity in the mutant was sequence-dependent by generating a DiffLogo (Figure 3). For the DiffLogo, reads mapping to the spacer regions were isolated, and the top 2.5% occupied positions were identified (consistent with previous publications [21,22,23,24,25]). In Figure 3, enriched sequences are included for *hmo1Δ* on top (JS divergence > 0), with WT underneath (JS divergence < 0). The last incorporated nucleotide (LNT) is indicated by the black arrow. These data demonstrate that in the *hmo1Δ* strain, Pol I is particularly sensitive to G-rich regions that are directly downstream of T-rich regions. Our findings suggest that Pol I pause sites are reorganized in the absence of *HMO1* and indicate that Hmo1 promotes efficient transcription elongation in a sequence-specific manner.

## 4. Discussion

Hmo1 has been implicated as an activator of transcription by Pol I for over two decades; however, the mechanism by which it promotes rRNA synthesis has remained unclear. Here, we used NET-seq to investigate the function of Hmo1 during rRNA synthesis in high-resolution detail. We found that in *hmo1Δ* yeast, Pol I is sensitive to altered pause sites, especially in G-rich regions of the 35 S gene, suggesting that Hmo1 may interact with topoisomerases to manage the topology of rDNA.

A recent publication demonstrated that Hmo1 may coordinate with and direct the binding of topoisomerases (especially Top2), which is essential for resolving supercoiling, thereby promoting efficient transcription via Pol II [18]. If Hmo1 plays a similar role in rDNA, we would expect that in *hmo1Δ* mutants, there would be a decrease in efficient transcription by Pol I, such as a reduction in the transcription elongation rate and/or an alteration to the pause profile of Pol I. While it is not possible to determine the elongation rate from NET-seq experiments, resultant polymerase occupancy patterns can predict regions of faster and slower elongation rates (the interpretation of peaks and valleys is discussed in detail in the Results Section 3.1 above). To this point, we observed that not only were there significant changes to the Pol I occupancy pattern in the *hmo1Δ* mutants, suggesting altered pausing, but that these alterations were exacerbated in the middle and at the 3′ end of the gene (Figure 1 and Figure 2). Altogether, our data demonstrate that Hmo1 has significant effects on Pol I occupancy and transcription elongation, as some pause sites were enhanced, whereas others were reduced in *hmo1Δ* yeast vs. WT. If Hmo1 is a direct enhancer of the elongation rate, we would expect a similar increase in occupancy across all pause sites. However, since this was not observed, our data support the prediction that Hmo1 could have a conserved function for both Pols I and II. In this case, it would be reasonable to expect that without Hmo1, supercoiling would be either partially resolved or resolved at a slower rate, leading to increased Pol I pausing that would worsen further in the transcribed region, which is exactly what our NET-seq data indicate (Figure 1 and Figure 2). Our data support this hypothesis, but further investigation is required to determine whether Hmo1 and Top2 maintain the topology of rDNA in the same way as Pol II-transcribed genes.

Our findings suggest that Pol I is significantly more prone to pausing, especially in T/G-rich regions, in *hmo1Δ* mutants compared to WT. Previously, it has been established that R-loops, structures containing a DNA/RNA hybrid and one displaced DNA strand, frequently form in rDNA [29] and that in yeast lacking *TOP1*, there is an accumulation of R-loops in this region [30]. Furthermore, it has been proposed that R-loops may form more readily in negatively supercoiled regions that are G-rich [31,32] and that these structures could cause polymerases to stall on the template [33] and even inhibit transcription elongation [34,35]. Therefore, we predict that perhaps the decreased efficiency of transcription in the *hmo1Δ* mutant could provide favorable conditions for R-loops to accumulate in rDNA, increasing Pol I pausing. This is consistent with our DiffLogo results, which indicate that Pol I pauses more frequently in G-rich regions of the 35 S gene in the *hmo1Δ* strain.

In yeast, Hmo1 has been proposed to be the functional analog to the human UBF protein, which is thought to be important for maintaining the euchromatic state of actively transcribed rDNA repeats. Yeast cells contain approximately 200 tandem rDNA repeats, where relatively half are actively transcribed by Pol I in a growing cell [36]. There is a lack of histones occupying these actively transcribed repeats, and instead, these regions are abundantly populated by Hmo1 [37]. The regulatory mechanisms that govern the maintenance of the rDNA chromatin state are still not well-defined, but it is reasonable to expect that transcription factors, such as Hmo1, could play a role in this process. In fact, in mammalian cells, when UBF levels are depleted, the number of active rDNA repeats is reduced [38]. Interestingly, this phenomenon was found to be reversible (the restoration of UBF levels caused a return to the baseline number of active repeats), suggesting that UBF directly regulates the chromatin state of rDNA in humans. Due to the previous study demonstrating that Hmo1 selectively associates with actively transcribed rDNA [37] and the sequence conservation and functional overlap between Hmo1 and UBF [11], we predict that Hmo1 could contribute to the maintenance of active rDNA repeats for Pol I in yeast.

In addition to the roles for Hmo1 proposed above, previous genetic studies further implicate this factor as an important component of Pol I machinery. Interestingly, *hmo1Δ* mutants that contained a secondary full deletion of either *RPA49* or *RPA12* or partial deletion of *RPA43* (*rpa43-12*) were determined to be synthetically lethal [10]. Furthermore, all single mutations (*hmo1Δ, rpa49Δ, rpa12Δ, rpa43-12*) were viable. These subunits interact with each other and Rpa49 and Rpa12, in particular, have been proposed to play various roles in transcription elongation [21,39,40,41]. We hypothesize that mutants that exhibit transcription elongation defects (such as *rpa49Δ* and *rpa12Δ*) could be hypersensitive to the altered pause profile caused by a deletion in *HMO1*, which is consistent with previous genetic studies.

It has long been proposed that Hmo1 is a component of Pol I machinery in yeast, but its mechanism of action remains unclear. Here, our results suggest that without Hmo1, Pol I occupancy is significantly repositioned on the rDNA template, especially in G-rich regions. These findings demonstrate that Hmo1 could play a conserved role in yeast and humans as a mediator of the rDNA chromatin state or that it could regulate transcription by Pols I and II by coordinating with topoisomerases. While indirect, these proposed mechanisms of action by Hmo1 are consistent with previous publications, proposed models for UBF function, and the high-resolution Pol I NET-seq results defined by this study.

## Figures and Tables

**Table 1:** Primer sequences for NET-seq library amplification for each sample.

Sample	Forward Primer	Reverse Primer
WT 1	CAAGCAGAAGACGGCATACGAGATttctgcctTCCGACGATCATTGATGGTGCC	AATGATACGGCGACCACCGAGATCTACACtagatcgcCGTCTCTTCTGCGGATGACTCG
WT 2	CAAGCAGAAGACGGCATACGAGATgctcaggaTCCGACGATCATTGATGGTGCC	AATGATACGGCGACCACCGAGATCTACACtagatcgcCGTCTCTTCTGCGGATGACTCG
WT 3	CAAGCAGAAGACGGCATACGAGATaggagtccTCCGACGATCATTGATGGTGCC	AATGATACGGCGACCACCGAGATCTACACtagatcgcCGTCTCTTCTGCGGATGACTCG
*hmo1Δ* 1	CAAGCAGAAGACGGCATACGAGATcatgcctaTCCGACGATCATTGATGGTGCC	AATGATACGGCGACCACCGAGATCTACACtagatcgcCGTCTCTTCTGCGGATGACTCG
*hmo1Δ* 2	CAAGCAGAAGACGGCATACGAGATgtagagagTCCGACGATCATTGATGGTGCC	AATGATACGGCGACCACCGAGATCTACACtagatcgcCGTCTCTTCTGCGGATGACTCG
*hmo1Δ* 3	CAAGCAGAAGACGGCATACGAGATcctctctgTCCGACGATCATTGATGGTGCC	AATGATACGGCGACCACCGAGATCTACACtagatcgcCGTCTCTTCTGCGGATGACTCG

**Table 2:** Software package versions used for NET-seq data analysis.

Software	Version
Anaconda	5.3.1
BEDTools	2.28.0
car	3.1-2
carData	3.0-5
cba	0.2-23
cowplot	1.1.1
cutadapt	3.4
dae	3.2.19
DiffLogo	2.14.0
dplyr	1.1.3
extrafont	0.19
ez	4.4-0
FastQC	0.11.7
forcats	1.0.0
fqtrim	0.9.7
ggforce	0.4.1
ggfortify	0.4.16
ggplot2	3.4.4
ggpubr	0.6.0
ggseqlogo	0.1
hexbin	1.28.3
matrixStats	0.63.0
pastecs	1.3.21
plyr	1.8.8
proxy	0.4-25
purrr	1.0.1
R	4.0.2
rclone	1.48.0
RcppRoll	0.3.0
readr	2.1.4
RStudio	1.3.959
SAMTools	1.6
scales	1.3.0
seqLogo	1.56.0
STAR	2.7.1a
statmod	1.4.36
stringr	1.5.1
tibble	3.2.1
tidyr	1.3.0
tweedie	2.3.5
zoo	1.8-12

## Data Availability

All raw and processed files are available in the Gene Expression Omnibus database with the WT files available at GSE216460 and the *hmo1Δ* files available at the GSE247981 accession. R scripts used to visualize the data and generate Figure 1, Figure 2 and Figure 3 and Supplementary Figure S1 are available upon request.

## Supplementary Materials

The following supporting information can be downloaded at: https://www.mdpi.com/article/10.3390/genes15020247/s1, Figure S1: Pol I NET-seq is reproducible across triplicate samples.
