# Supplementary material for: Hmo1 Promotes Efficient Transcription Elongation by RNA Polymerase I in Saccharomyces cerevisiae
Source: Genes (Basel). 2024 Feb 15;15(2):247. doi: 10.3390/genes15020247 (PMC10888141; doi:10.3390/genes15020247)
Supplement: Supplementary file 1 [file genes-15-00247-s001.zip › genes-2841462-supplementary.pdf]

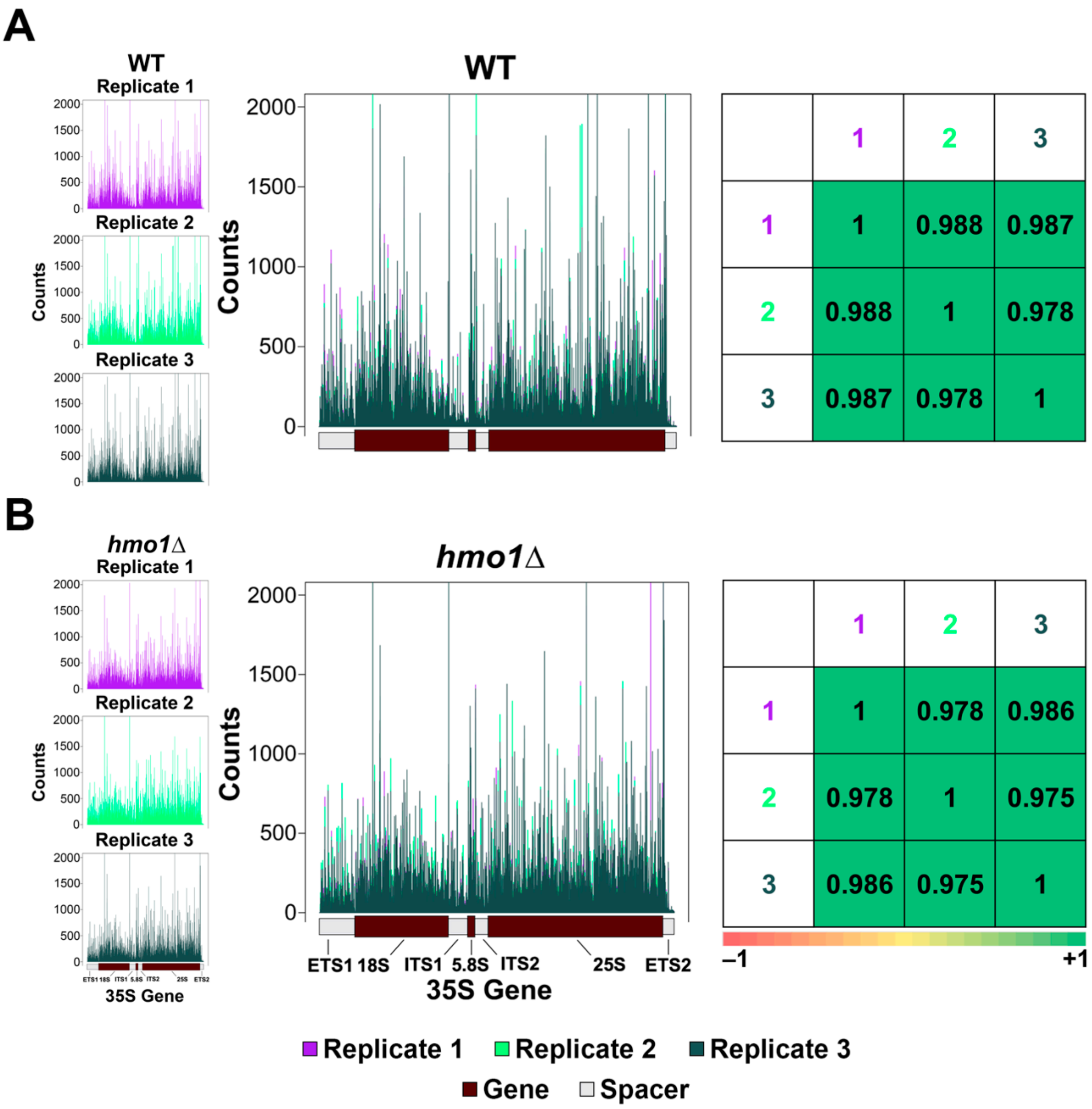


**A** (WT)

| | 1 | 2 | 3 |
|---|---|---|---|
| 1 | 1 | 0.988 | 0.987 |
| 2 | 0.988 | 1 | 0.978 |
| 3 | 0.987 | 0.978 | 1 |

**B** (*hmo1Δ*)

| | 1 | 2 | 3 |
|---|---|---|---|
| 1 | 1 | 0.978 | 0.986 |
| 2 | 0.978 | 1 | 0.975 |
| 3 | 0.986 | 0.975 | 1 |

**Supplemental Figure S1. Pol I NET-seq is reproducible across triplicate samples.** After alignment, reads mapping to the rDNA were isolated, normalized (as described in the methods and the results (3.1) sections), and plotted for the (A) WT and (B) *hmo1Δ* strains. Histograms were generated for each of the three separate replicates (left panels) and with all replicates overlaid (middle panels), where “counts” represents the number of polymerases at that single-nucleotide position on the rDNA at the time of harvest. Spearman correlation coefficient values (where 1 represents total similarity between two replicates) are included in the right panels for the comparison between one replicate vs. each of the other two within either the WT or the *hmo1Δ* strain.
